# Supplementary material for: Estimating total human deaths from rabies: A statistical framework leveraging reports of rabies transmission from deceased organ and tissue donors
Source: PLoS Negl Trop Dis. 2026 Sep 21;20(9):e0014731. doi: 10.1371/journal.pntd.0014731 (PMC13592719; doi:10.1371/journal.pntd.0014731)
Supplement: Supplementary file 6 [file pntd.0014731.s005.zip › Result_B/the_US/Figure_S3_the_US.pdf]

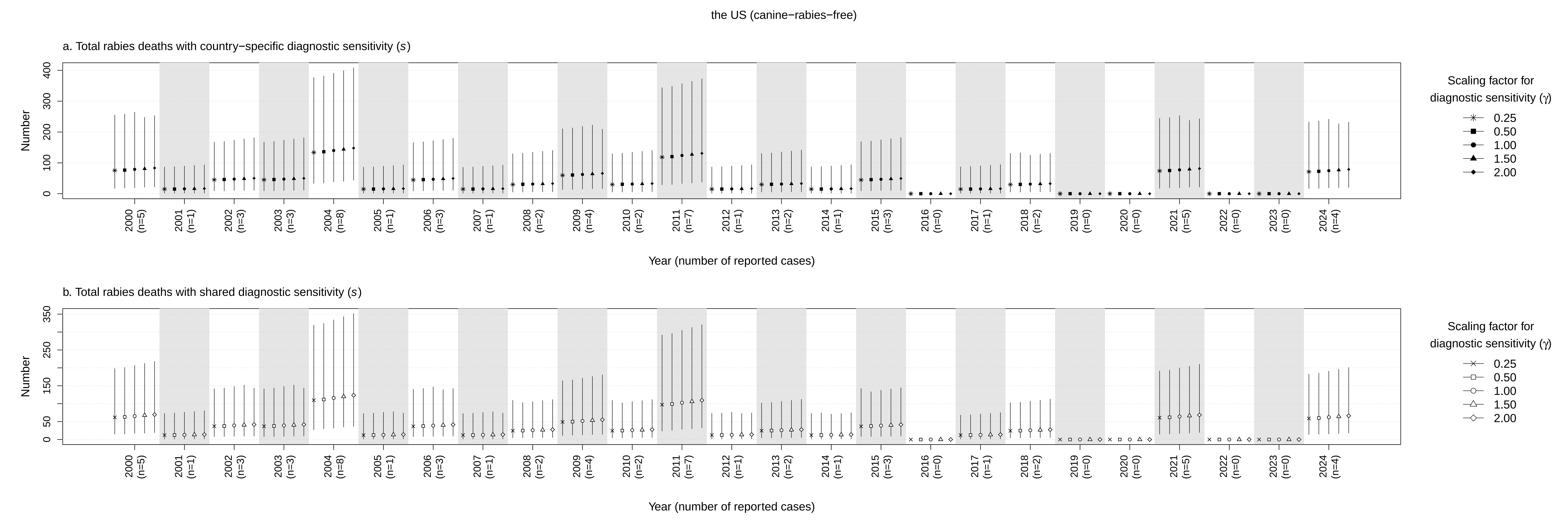

the US (canine−rabies−free)
a. Total rabies deaths with country−specific diagnostic sensitivity (s)
b. Total rabies deaths with shared diagnostic sensitivity (s)
Number
0
100
200
300
400
50
150
250
350
2000 (n=5)
2001 (n=1)
2002 (n=3)
2003 (n=3)
2004 (n=8)
2005 (n=1)
2006 (n=3)
2007 (n=1)
2008 (n=2)
2009 (n=4)
2010 (n=2)
2011 (n=7)
2012 (n=1)
2013 (n=2)
2014 (n=1)
2015 (n=3)
2016 (n=0)
2017 (n=1)
2018 (n=2)
2019 (n=0)
2020 (n=0)
2021 (n=5)
2022 (n=0)
2023 (n=0)
2024 (n=4)
Year (number of reported cases)
Scaling factor for diagnostic sensitivity (γ)
0.25
0.50
1.00
1.50
2.00
